# Transoral robotic surgery for parapharyngeal carcinoma ex‐pleomorphic adenoma: A case report

**DOI:** 10.1002/cnr2.1692

**Published:** 2022-08-09

**Authors:** Enrique Cadena‐Piñeros, Andrey Moreno‐Torres, Jessica Correa‐Marin, Mario A. Melo‐Uribe

**Affiliations:** ^1^ Department of Head and Neck Instituto Nacional de Cancerología Bogotá DC Colombia; ^2^ Department of Otorhinolaryngology Universidad Nacional de Colombia and Hospital Universitario Nacional de Colombia Bogotá DC Colombia; ^3^ Department of Surgery Cínica Country Bogotá DC Colombia; ^4^ Department of Pathology Instituto Nacional de Cancerología Bogotá DC Colombia; ^5^ Department of Pathology Fundación Universitaria de Ciencias de la Salud Bogotá DC Colombia

**Keywords:** parapharyngeal space, pleomorphic adenoma, robotic surgery, transoral, transoral robotic surgery

## Abstract

**Background:**

Carcinoma ex‐pleomorphic adenoma (Ca ex‐PA) comprises 0.5% of head and neck neoplasms. Transoral robotic surgery (TORS) is an approach being used to treat a variety of benign and malignant head and neck neoplasms. Recently, this technique has gained popularity as an alternative for parapharyngeal space (PPS) tumor resection. To our knowledge, this is the first case of Ca ex‐PA managed successfully by TORS of the PPS.

**Case:**

Fifty‐nine‐year‐old male with incidental mass in PPS, initial diagnosis of pleomorphic adenoma, who underwent transoral robotic resection. The histopathology diagnosis with minimally invasive Ca ex‐PA findings and malignant component of high‐grade epithelial/myoepithelial carcinoma and salivary duct carcinoma. Patient discharged on the fifth post‐operative day without complications.

**Conclusion:**

Based on our findings, TORS may be a safe procedure to remove selected Ca ex‐PA from the PPS; however, further research is needed.

## 1 INTRODUCTION

Carcinoma ex‐pleomorphic adenoma (Ca ex‐PA) is a rare, aggressive, and poorly differentiated malignant neoplasm, representing only 0.5% of head and neck neoplasms.^1^ It is defined as a carcinoma arising from the primary (de novo) or recurrent benign pleomorphic adenoma in a salivary gland.^2^ The histopathological diagnosis is based on the coexistence of epithelial malignancy with histologically benign pleomorphic adenoma. It predominantly affects the major salivary glands with peak incidence occurring on the sixth decade of life and a slight female predominance. The prognosis for Ca‐ex‐PA depends on the stage, histologic grade, and local infiltration. Patients with minimally invasive tumors have excellent prognosis.^3^, ^4^

The presentation of Ca ex‐PA in the parapharyngeal space (PPS) is around 3% and is located mainly in the prestyloid area.^5^ PPS tumors are most often treated by surgical excision. Its treatment can be challenging for the head and neck surgeon; however, there are multiple surgical approaches including transoral, transcervical, orbitozygomatic, transcervical with mandibulotomy and combined transoral and transcervical approaches.^6^, ^7^ Major surgeries are required to fully resect parapharyngeal space tumors. Due to the relative inaccessibility of PPS and the proximity of neoplasms to important neurovascular structures, transcervical surgical approaches are regularly recommended.^8^ In recent years, transoral robotic surgery (TORS) has generated great enthusiasm for the treatment of benign and malignant tumors of the oropharynx,^9^, ^10^, ^11^ and its use has expanded to lesions in the larynx, cranial floor, nasopharynx and the parapharyngeal space.^8^ TORS is a minimally invasive procedure that can represent an alternative to open surgery^11^, ^12^ in a few cases, as the one described in this article.

We are describing a case of a 59‐year‐old male diagnosed with Ca ex‐PA, developed in the PPS. The tumor was resected completely by TORS. The outcome reflected that this technique allows for easily achieved 3D visualization of the tumor in the PPS, shorter operative times, increase in range of motion in the narrow operative field, and a complete pathological specimen removal. Our literature review suggests that this is the first case of Ca ex‐PA of PPS successfully managed by the TORS.

## 2 CASE REPORT

A 59‐year‐old man arrived at a rural hospital in Colombia in November, 2020 after sustaining a scorpion bite on the left foot and was treated accordantly. The patient developed edema and swelling on the left side of the neck; he was treated for cervical suppurative adenitis with surgery and antibiotics.

**FIGURE 1:**
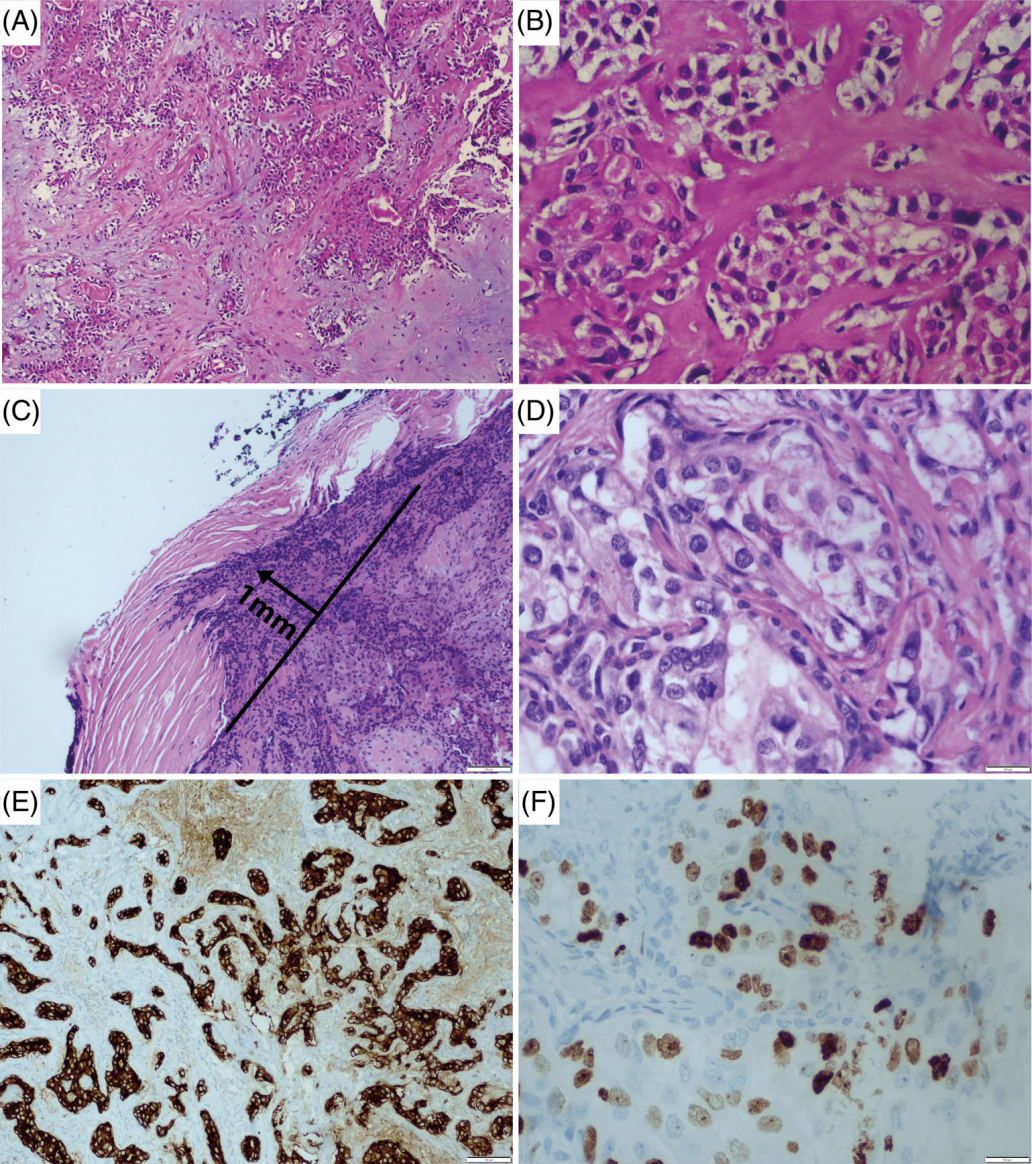
Hematoxylin & eosin (H&E) staining. (A, B) (H&E) Initial biopsy benign tumor pleomorphic adenoma 10× and 40×, respectively. (C) (H&E, 10×) The neoplastic infiltration of the lesion capsule is observed with 1 linear mm of tumor invasion. (D) (H&E, 40×) The malignant component of epithelial/myoepithelial carcinoma is identified. (E) (Immunohistochemical Pathology 10×) Positivity for epithelial membrane antigen (EMA) is identified. (F) (40×) A Ki67 cell proliferation index of 30% is observed

During the post op follow up 1 month later, a CT scan of the neck was obtained reflecting incidental findings of a mass in the right PPS followed by a CT‐guided biopsy. The pathology report indicated epithelial/myoepithelial neoplasia compatible with pleomorphic adenoma (Figure 1A,B). With these findings, the patient was referred to the Head and Neck Surgery Department of the National Cancer Institute in Bogotá‐Colombia for treatment.

**FIGURE 2:**
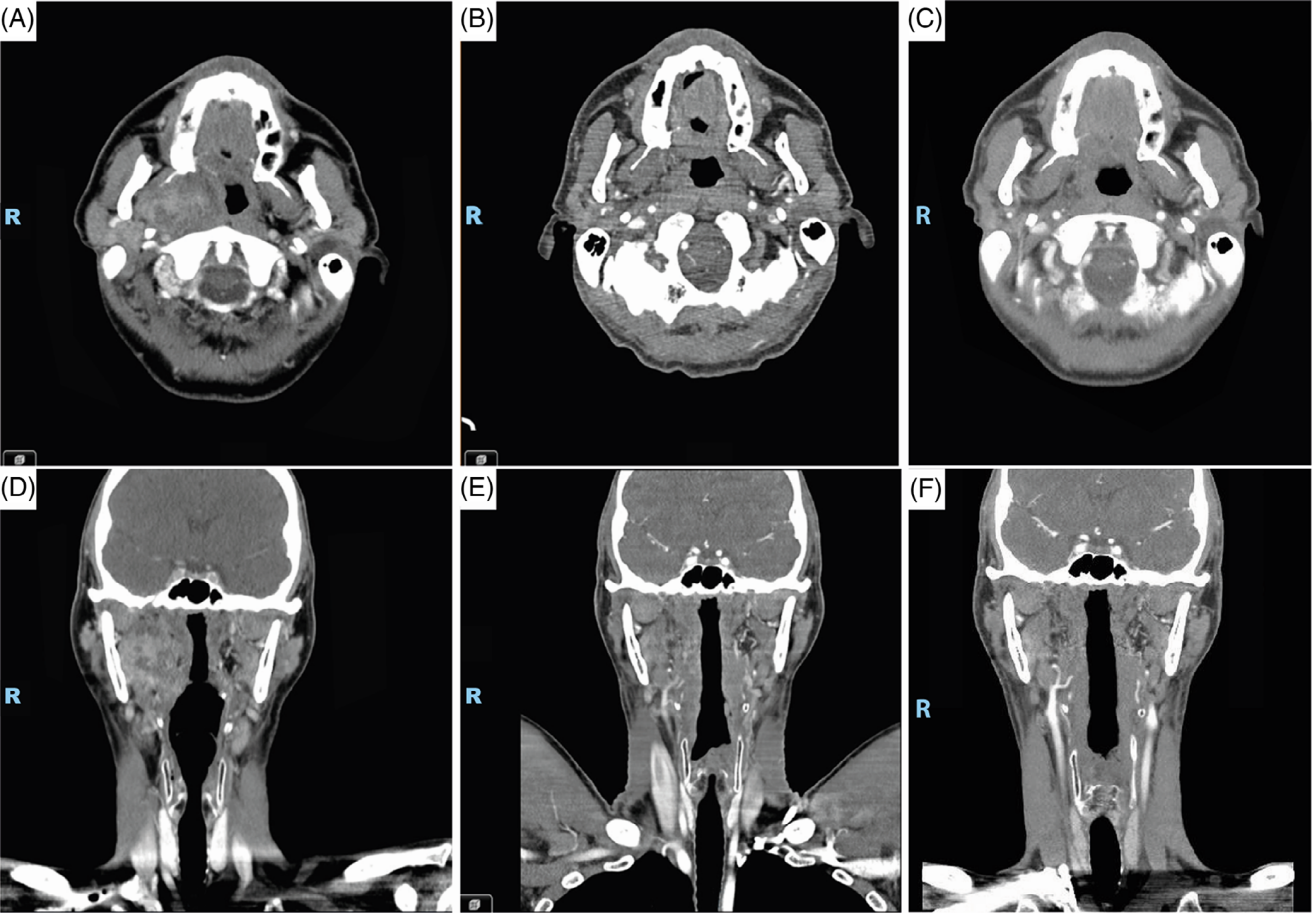
Pre‐surgical CT scan. 40 × 29 mm lesion in the right parapharyngeal space: (A) axial and (D) coronal images. Post‐surgical CT scan, follow‐up after 8 weeks of treatment: (B) axial and (E) coronal images. Follow‐up after 7 months of treatment: (C) axial and (F) coronal images

A new neck CT scan was taken upon arrival revealing a 40 × 29 mm rounded mass located in the prestyloid space (Figure 2A,C). Flexible fiberoptic laryngoscopy ruled out nasopharyngeal extension.

After a thorough review of the biopsy by an oncologic pathologist, a medical board meeting was held at which it was concluded to be a benign pleomorphic adenoma; it was decided to perform robot‐assisted transoral resection in July 2021.

### 2.1 Surgical technique

#### 2.1.1 TORS procedure for a transoral resection of the PPS tumor

**FIGURE 3:**
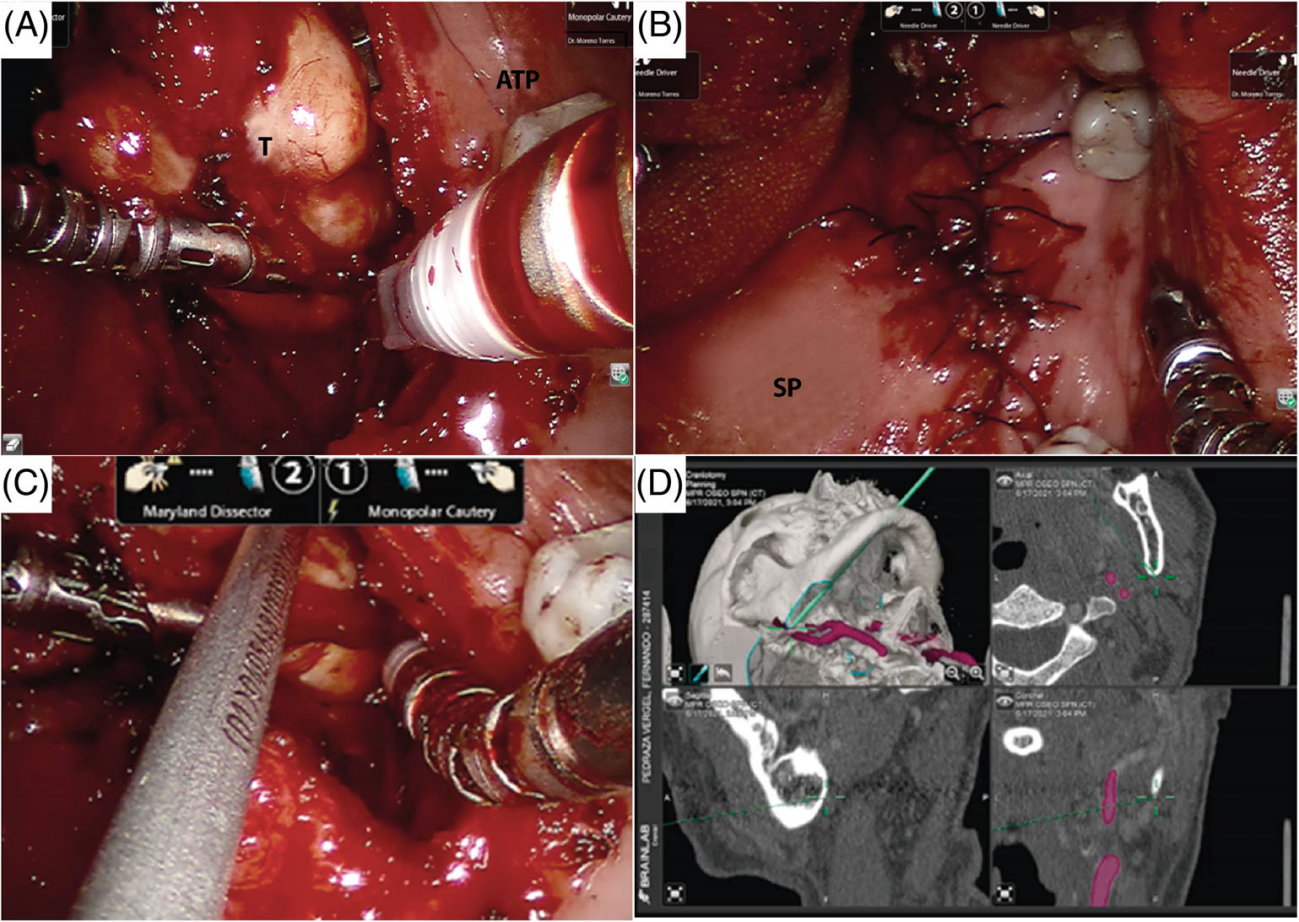
Images obtained using the TORS technique. (A) Resection of the parapharyngeal space mass, ATP (anterior tonsillar pillar)/T (tumor). (B) Primary closure of the defect (soft palate). (C, D) Intraoperative navigation: internal carotid artery (red)

Under general anesthesia and after nasotracheal intubation, the da Vinci robotic® (Intuitive Surgical, INC., Sunnyvale, CA, USA) system was docked above the patient's head. Then, a Crowe‐Davis mouth gag, 8 mm HD telescopic camera 0° and 5 mm EndoWrist® instruments were placed. The surgical assistant located at the head of the table performed suction, retraction, and the operating physician sat at the surgeon's console. After conventional right tonsillectomy, a transfixion medial incision was made between the anterior and posterior tonsil pillars, being the superior constrictor muscle the upper edge; the PPS was acceded, and the tumor was identified (Figure 3A). A Brainlab neuro navigator and a Hitachi ultrasound endoscopic transducer were used to identify the neovascular structures of the PPS. Complete tumor resection was achieved without capsule spill, and no complications at the end of the procedure (Figure 3B–D). The patient was extubated and transferred to the general ward: oral intake was resumed after 5 days, and he was discharged without postoperative complications.

Surgical pathology demonstrated a minimally invasive carcinoma, 2.8 × 1.7 × 0.4 cm ex‐pleomorphic adenoma with a malignant component of high‐grade epithelial/myoepithelial carcinoma and salivary duct carcinoma. Microscopic examination showed the presence of necrosis and mitotic activity of at least 7 mitoses in 10 high‐power fields. No lymphovascular invasion or perineural invasion was observed; the resection edges were negative, with neoplastic infiltration of the lesion capsule with 1 linear mm of tumor invasion (Figure 1C,D). Immunohistochemical results were positive for ck7, p63, CEA, EMA, p40, and androgens. A Ki67 cell proliferation index of 30% was also found (Figure 1E,F). Further imaging was negative for metastases in all instances (Brain, thoracic, and abdominal CT scans).

Due to the high‐risk tumor reported, he underwent adjuvant IMRT technique external radiotherapy with 1.8 Gy to 54 Gy per fraction in low‐risk volume (tumor bed and bilateral elective drainage) and simultaneous integrated reinforcement in a 2.2 to 66 Gy per fraction in high‐risk volume (tumor bed) ending in November 2021. A follow up image was taken 8 weeks later, without tumor persistence (Figure 2B,D). The patient was scheduled for routine follow ups every 3 months; on his second follow up he remained without swallowing dysfunction nor pain; the follow up CT was interpreted as disease‐free (Figure 2E,F).

## 3 DISCUSSION

The surgical treatment method for this patient was TORS technique, considering the multiple studies that have reported its advantages in the treatment of benign and malignant tumors of the oropharynx and larynx,^8^, ^9^, ^10^, ^11^, ^12^, ^13^, ^14^ and its low morbidity rate.^9^, ^13^ Its indications for the management of resections of benign tumors like adenoma pleomorphic of the PPS are known.^15^ However, its usage continues to be controversial for malignant tumors of this location.^11^ Critics of this approach do not favor its usage, arguing that there is limited exposure, especially for larger tumors increasing the risk for both tumor spillage/implantation and the risk of infection and neurovascular injury even with careful pre‐ operative evaluation.^11^

Currently, in the literature there are 32 PPS Ca ex‐PA reported cases,^16^ all these managed through open approaches; nevertheless, the recurrence rate is high, mainly in high‐risk tumors^7^ due to their aggressive behavior, which will predominantly depend on their histopathologic features.

The surgical approach depends on tumor localization, size, initial biopsy, and compromise of adjacent structures by the neoplasia. Patients with PPS tumors must meet the following criteria to be considered as candidates for surgical removal with TORS approach at our institution: have well‐circumscribed tumors up to 8 cm in size,^17^ projecting into the oropharynx even with poststyloid involvement and, laterally displacing the internal carotid artery^18^; in addition, the presence of trismus in patients should be evaluated, as it may limit intraoral exposure and proper placement of robotic tools.^17^, ^18^

The importance of studying radiological images before surgery guides not only the question of resectability but the surgical approach to be used.^6^, ^9^, ^11^, ^17^ In this case, the CT scan allowed visualization of a small tumor in the prestyloid area without contact to the internal carotid artery or lower cranial nerves deemed suitable for resection with the TORS approach according to our standards. Initial biopsy was interpreted as an adenoma pleomorphic tumor. Sensitivity of fine needle aspiration biopsy (FNAB) in the diagnosis of Ca ex‐PA is 64%–92% and its specificity is 86%–98% although this result can be affected by the sample.^16^ In our experience, if the initial biopsy had been reported as a malignant tumor, we would have also indicated resection by TORS, with radical resection of the oropharyngeal wall with reconstruction of the lateral defect of the oropharynx with a free flap.^10^

When a patient is suitable for transoral approach, the preferred method at our institution is TORS over transoral endoscopic surgery, as it not only allows greater movement in such a narrow working space, but also provides more precision in achieving resection margins due to its wide near‐field visualization.

There is lack of information regarding oncological results in this type of tumors, given the rarity of Ca ex‐PA, and in the vast majority of cases its identification is in the surgical specimen, after surgeries without oncologic intention and very conservative.From what has been described in retrospective series in parotid tumors, resections with extracapsular dissection in Ca ex‐PA with minimal capsular infiltration or low‐grade tumor seem to be sufficient without affecting survival or postoperative morbidity.^19^ In our case, despite having negative resection margins, minimal capsular infiltration and no spillage, it presented other pathological characteristics that led to the determination of a high‐grade tumor thus, the concept of the multidisciplinary group was adjuvant with IMRT.

Traditional open methods, in contrast to minimally invasive approaches such as TORS, have the disadvantage of requiring external incisions and extensive dissection of unaffected anatomical structures.^20^ One of the main difficulties in using TORS is the complex transoral anatomy of the oropharynx and parapharyngeal spaces, so the surgeon must master the inside‐out anatomy of these spaces to perform transoral robotic surgery in a safe and efficiently manner.^13^

Recently, transoral approaches have undergone a complete renovation with robotic surgery since it has great advantages such as a wide near‐field visualization, providing excellent image quality.^9^ The increase in the scale of movement and the degree of freedom of TORS are particularly useful when access is limited, as it is in the case of the PPS.^9^ Therefore, it makes an impact in decreasing the length of the hospitalization, less blood loss, and postoperative pain, as well as the intact preservation of facial appearance.^9^, ^10^

However, there are some key considerations to keep in mind for the TORS approach to be contraindicated: if the tumor appears infiltrative radiographically, has a poorly defined plane with respect to the great vessels, or displaces the internal carotid medially; in these cases, a transcervical approach should be considered.^17^

According to the guideline recommendations on the adjuvance in malignancy of the salivary glands,^21^ patients with risk factors for locoregional relapse, such as capsular invasion, tumor size, advanced stage, positive margins after primary surgical treatment, and perineural and vascular invasion,^22^ should receive radiotherapy, regardless of their approach. In this case, a mitotic activity of at least 7 mitoses were found in 10 high‐power fields, necrosis, and the neoplastic infiltration of the lesion capsule was observed, so, IMRT was administered to our patient.

## 4 CONCLUSION

Based on the results obtained in this case, we have achieved an effective approach for the resection of a PPS located Ca ex‐PA case without complications. The wide near‐field 3D visualization, shorter operative times and, greater range of motion in narrow spaces are some of the advantages of this technique that contributed to its success while also minimizing bleeding, and avoiding morbid open surgery; as far as limitations we can point out a piece of equipment that is not always available (the da Vinci robotic®) and a prolonged surgical exposure to its use by surgeons to improve results. To our knowledge, this is the first documented case of minimally invasive surgery in Ca ex‐PA by TORS; this is a promising technique, though further studies are still needed.

## AUTHOR CONTRIBUTIONS

**Enrique Cadena‐Piñeros:** Conceptualization (equal); data curation (equal); formal analysis (equal); investigation (equal); methodology (equal); resources (equal); supervision (equal); validation (equal); visualization (equal); writing – original draft (equal); writing – review and editing (equal). **Andrey Moreno‐Torres:** Data curation (equal); validation (equal); visualization (equal); writing – review and editing (equal). **Jessica Correa‐Marin:** Conceptualization (equal); data curation (equal); formal analysis (equal); investigation (equal); methodology (equal); project administration (equal); resources (equal); software (equal); supervision (equal); validation (equal); visualization (equal); writing – original draft (equal); writing – review and editing (equal). **Mario Alexander Melo‐Uribe:** Validation (equal); writing – original draft (equal); writing – review and editing (equal).

## FUNDING INFORMATION

This research did not receive any specific grants from funding agencies in the public, commercial, or not‐for‐profit sectors.

## CONFLICT OF INTEREST

The authors have stated explicitly that there are no conflicts of interest in connection with this article.

## ETHICS STATEMENT

Authors confirm that all procedures followed, were in accordance with the ethical standards with the Helsinki declaration of 1975, as revised in 2000.

## PATIENT CONSENT STATEMENT

Informed consent was obtained from the patient for the publication of the details of his case and the use of clinical and personal images.

## Data Availability

Data sharing is not applicable to this article as no new data were created or analyzed in this study.
